# Some Historic Children's Hospital Cots

**Published:** 1914-06-06

**Authors:** 


*To the Editor of* THE HOSPITAL.

### Some Historic Children's Hospital Cots.

*To the Editor of* THE HOSPITAL.

SIR,—In THE HOSPITAL of May 23 there was an interesting article on "Some Historic Children's Hospital Cots." It may be of interest to read this week of the "Princess May" Cot at the Royal Alexandra Hospital, Rhyl, which was founded and endowed in 1893 by ladies in the county of Shropshire as a wedding gift to Queen Mary (then Duchess of York).

Children from the county of Shropshire have a prior claim to this cot, the occupants of which are always nominated by the Queen. The cot is invaluable to children needing very long treatment at the seaside, who otherwise could not possibly remain in the hospital till they were well. One little boy suffering from very bad spine disease occupied the cot for three years, and returned home quite well a short time ago, to the great delight of his family. His place has been filled by a little boy with acute hip trouble.—Yours truly,

LADY SUPERINTENDENT.

